# Lack of Effect of Lowering LDL Cholesterol on Cancer: Meta-Analysis of Individual Data from 175,000 People in 27 Randomised Trials of Statin Therapy

**DOI:** 10.1371/journal.pone.0029849

**Published:** 2012-01-19

**Authors:** Cholesterol Treatment Trialists' (CTT) Collaboration

**Affiliations:** Innsbruck Medical University, Austria

## Abstract

**Background:**

Statin therapy reduces the risk of occlusive vascular events, but uncertainty remains about potential effects on cancer. We sought to provide a detailed assessment of any effects on cancer of lowering LDL cholesterol (LDL-C) with a statin using individual patient records from 175,000 patients in 27 large-scale statin trials.

**Methods and Findings:**

Individual records of 134,537 participants in 22 randomised trials of statin versus control (median duration 4.8 years) and 39,612 participants in 5 trials of more intensive versus less intensive statin therapy (median duration 5.1 years) were obtained. Reducing LDL-C with a statin for about 5 years had no effect on newly diagnosed cancer or on death from such cancers in either the trials of statin versus control (cancer incidence: 3755 [1.4% per year [py]] versus 3738 [1.4% py], RR 1.00 [95% CI 0.96-1.05]; cancer mortality: 1365 [0.5% py] versus 1358 [0.5% py], RR 1.00 [95% CI 0.93–1.08]) or in the trials of more versus less statin (cancer incidence: 1466 [1.6% py] vs 1472 [1.6% py], RR 1.00 [95% CI 0.93–1.07]; cancer mortality: 447 [0.5% py] versus 481 [0.5% py], RR 0.93 [95% CI 0.82–1.06]). Moreover, there was no evidence of any effect of reducing LDL-C with statin therapy on cancer incidence or mortality at any of 23 individual categories of sites, with increasing years of treatment, for any individual statin, or in any given subgroup. In particular, among individuals with low baseline LDL-C (<2 mmol/L), there was no evidence that further LDL-C reduction (from about 1.7 to 1.3 mmol/L) increased cancer risk (381 [1.6% py] versus 408 [1.7% py]; RR 0.92 [99% CI 0.76–1.10]).

**Conclusions:**

In 27 randomised trials, a median of five years of statin therapy had no effect on the incidence of, or mortality from, any type of cancer (or the aggregate of all cancer).

## Introduction

Randomised trials have shown that lowering low density lipoprotein (LDL) cholesterol with a statin substantially reduces the risk of major vascular events in a wide range of people [1], and that further reductions in LDL cholesterol with more intensive statin regimens produce further reductions in risk [2]. Statins are able to lower LDL cholesterol to well below 2 mmol/L (80 mg/dL) in many individuals, and LDL cholesterol concentrations as low as this have been associated with an excess risk of cancer in observational cohort studies [3]. Such associations have generally been attributed to reverse causality arising from the tendency for undetected cancers to lower LDL cholesterol [4]; [5]. The availability of a large number of cancers in randomised trials of statins now allow an unbiased assessment of whether reducing LDL cholesterol with a statin causes cancer.

Although several published tabular meta-analyses of randomised trials involving large numbers of cancers indicate that standard statin regimens do not increase the aggregate risk of any cancer over a period of around 4–5 years [6]–[9], such analyses are unable to address concerns that lowering LDL cholesterol with a statin might increase the risk of particular types of cancer. This possibility had originally been raised by the results of individual statin trials. For example, apparent excesses of gastrointestinal cancer in the PROSPER trial [10] and breast cancer in the CARE trial [11] generated considerable concern about the safety of statins, despite a lack of corroborating evidence from other trials [1]. Moreover, because patients in PROSPER were aged 70 or over, it was suggested that there might be an excess risk of cancer among elderly people.

The Cholesterol Treatment Trialists' (CTT) Collaboration has recently reported analyses of the effects on major clinical outcomes of further reductions in LDL cholesterol resulting from more intensive statin regimens, and updated analyses of the effects of standard statin regimens [2]. That meta-analysis concluded that there was no overall evidence of any excess risk of cancer, or of cancer mortality, associated with statin therapy. However, there is a need for a more detailed assessment of specific types of cancer to determine whether lowering LDL cholesterol with statins might increase or decrease the risk of various cancers, as well as a need for a more detailed assessment of whether lowering LDL cholesterol to very low concentrations might increase the risk of cancer. The present report, which includes individual patient data on over 10,000 cancers among 175 000 participants in 27 statin trials (including one trial not available in the previous analysis [12] and 5 trials involving assessment of more-intensive LDL-lowering therapy [13]–[17]) aims to provide such an assessment.

## Methods

The methods of the Cholesterol Treatment Trialists' Collaboration have been described in detail previously [1]; [2]; [18]. Trials were eligible for inclusion if: (i) the main effect of the intervention was to lower LDL cholesterol; (ii) no other differences in risk factor modification were intended; and (iii) at least 1000 participants were to be recruited with at least 2 years' treatment duration [2]. Each trial supplied individual patient data which were checked centrally, recoded into a standard format for analysis, summarized and verified for accuracy by the trialists.

The current analyses are of the incidence of cancer and of death from cancer. Cancers were coded using the 9^th^ revision of the International Classification of Disease (ICD-9), and subdivided into 23 detailed and 7 broad categories of sites (Table S1): gastrointestinal (ICD9 140-159); genitourinary (179–189); respiratory (160–163; 165); female breast (174), haematological (200–208), melanoma (172); and other specified or unspecified sites (other codes in ICD-9 140–209). Nonfatal non-melanoma skin cancers (173), benign neoplasms (210–229), cancers in situ (230–234) and neoplasms of uncertain (235–238) or unspecified (239) nature were excluded, as were nonfatal cancers known to be recurrences of primary tumours diagnosed prior to randomization and deaths from such recurrences. (During the detailed coding process undertaken for these analyses, minor corrections to previously published results [2] were made for several of the trials.) The main planned analyses were the effects of statin therapy on specific categories of primary cancers, and on cancer incidence (and cancer death) subdivided by year of follow-up, baseline LDL cholesterol, age, sex and other baseline characteristics.

### Statistical Methods

Analyses were to include all randomised patients irrespective of whether they received their allocated treatment (“intention-to-treat”). The primary meta-analyses were of the effects on cancer event rates in each trial calculated as the logrank (*o–e*) and its variance (*v*) for first events [2]. Analyses were performed both weighted and unweighted for the absolute LDL cholesterol difference in each trial at one year (*d* mmol/L) [2]. In a meta-analysis of several trials, the log of the rate ratio per mmol/L (log RR) is calculated as S/V with variance 1/V (and hence with 95% CI of S/V±1·96/√V), where S is the sum over all trials of *d (o–e)* and V is the sum over all trials of *d^2^v*. (For unweighted analyses, *d* is omitted from these formulae.) In subgroup analyses by baseline LDL cholesterol concentration, the *relevant* baseline lipid values in the trials comparing more versus less intensive statin therapy are those achieved on the less intensive regimen. However, in 3 of these trials [13]; [15]; [16], statin therapy was stopped before randomization, so the values at randomization (i.e. off statin treatment) tend to be overestimates of the relevant values. The relevant baseline values for these 3 trials were therefore estimated by multiplying the values at randomization by the mean proportional reduction in LDL cholesterol observed at one year among those allocated the less intensive regimen. Proportional risk reductions in different subgroups were compared by standard χ^2^ tests for heterogeneity or, where appropriate, trend. To help allow for multiple subdivisions, only summary rate ratios (indicated by open diamonds in figures) have 95% confidence intervals (CIs); all other rate ratios have 99% CIs. Analyses were done using SAS version 9.2 (SAS institute, Cary) and R version 2.11.1 (www.R-project.org)

## Results

Individual participant data were available from 27 trials of statin therapy involving 174 149 participants (22 trials of statin versus control [including one trial, CORONA [12], not previously available for the second analysis cycle [2]] and 5 trials of more versus less statin) (Table 1). (Individual participant data were unavailable for these analyses from just 2 eligible trials involving 6331 participants: SPARCL [19], and GREACE [20].) For the meta-analyses of statin versus control, the mean baseline LDL cholesterol was 3.70 mmol/L, the mean LDL cholesterol difference at one year was 1.08 mmol/L, and the median follow-up duration among survivors was 4.8 years. For the meta-analyses of more versus less intensive statin therapy, the weighted mean baseline LDL cholesterol was 2.53 mmol/L, the weighted mean LDL cholesterol difference at one year was 0.51 mmol/L, and the weighted median follow-up duration among survivors was 5.1 years.

**Table 1:** Baseline characteristics and eligibility criteria of participating trials.

	Number of patients	Treatment comparison (mg per day)	Median follow-up in survivors (years)*	Mean age (years)	Baseline LDL-C (mmol/L)	Prior CHD (%)**	Other vascular disease (%)†	No prior vascular disease (%)	Women (%)	LDL-C difference at 1 year (mmol/L)
**Statin vs. control**										
SSSS	4,444	S20-40 vs. placebo	5.4	59	4.88	4,444 (100%)	126 (3%)	0 (0%)	827 (19%)	−1.77
WOSCOPS	6,595	P40 vs. placebo	4.8	55	4.96	338 (5%)	193 (3%)	6,096 (92%)	0 (0%)	−1.07
CARE	4,159	P40 vs. placebo	5.0	59	3.58	4,159 (100%)	0 (0%)	0 (0%)	576 (14%)	−1.03
Post CABG	1,351	L40-80 vs. L2.5-5	4.3	61	4.02	1,351 (100%)	37 (3%)	0 (0%)	102 (8%)	−1.07
AFCAPS/TexCaps	6,605	L20-40 vs. placebo	5.2	58	3.89	10 (<1%)	9 (0%)	6,586(>99%)	997 (15%)	−0.94
LIPID	9,014	P40 vs. placebo	6.0	61	3.88	9,014 (100%)	905 (10%)	0 (0%)	1,516 (17%)	−1.03
GISSI-P	4,271	P20 vs. no treatment	2.0	59	3.92	4,271 (100%)	179 (4%)	0 (0%)	587 (14%)	−0.35
LIPS	1,677	F80 vs. placebo	3.9	60	3.42	1,677 (100%)	142 (8%)	0 (0%)	271 (16%)	−0.92
HPS	20,536	S40 vs. placebo	5.4	63	3.38	13,386 (65%)	8,865 (43%)	3,161 (15%)	5,082 (25%)	−1.29
PROSPER	5,804	P40 vs. placebo	3.3	75	3.79	1,881 (32%)	1,026 (18%)	3,254 (56%)	3,000 (52%)	−1.04
ALLHAT-LLT	10,355	P40 vs. usual care	4.9	67	3.76	1,188 (11%)	1,788 (17%)	8,037 (78%)	5,051 (49%)	−0.54
ASCOT-LLA	10,305	A10 vs. placebo	3.3	63	3.44	15 (<1%)	1,435 (14%)	8,860 (86%)	1,942 (19%)	−1.07
ALERT	2,102	F40 vs. placebo	5.5	50	4.14	400 (19%)	241 (11%)	1,702 (81%)	715 (34%)	−0.84
CARDS	2,838	A10 vs. placebo	4.1	62	3.03	9 (<1%)	97 (3%)	2,738 (96%)	909 (32%)	−1.14
ALLIANCE	2,442	A10-80 vs. usual care	4.7	61	3.80	2,442 (100%)	162 (7%)	0 (0%)	434 (18%)	−1.16
4D	1,255	A20 vs. placebo	4.0	66	3.25	630 (50%)	666 (53%)	344 (27%)	578 (46%)	−0.89
ASPEN	2,410	A10 vs. placebo	4.0	61	2.93	578 (24%)	302 (13%)	1,663 (69%)	811 (34%)	−0.99
MEGA † †	8,214	P10-20 vs. usual care	5.0	58	4.05	42 (<1%)	53 (1%)	8,119 (99%)	5,547 (68%)	−0.67
JUPITER	17,802	R20 vs. placebo	2.0	66	2.70	0 (0%)	0 (0%)	17,802 (100%)	6,801 (38%)	−1.09
GISSI-HF	4,574	R10 vs. placebo	4.2	67	3.06	1,797 (39%)	4,574 (100%)	0 (0%)	1,032 (23%)	−0.92
AURORA	2,773	R10 vs. placebo	4.6	64	2.58	659 (24%)	743 (27%)	1,663 (60%)	1,050 (38%)	−0.99
CORONA	5,011	R10 vs. placebo	3.0	73	3.55	4,377 (87%)	5,011 (100%)	0 (0%)	1,180 (24%)	−1.19
**Subtotal (22 trials)**	**134,537**	**-**	**4.8||**	**63||**	**3.70||**	**52,668 (39%)**	**26,554 (20%)**	**70,025 (52%)**	**39,008 (29%)**	**-1.08||**
**More vs. less statin**										
PROVE-IT	4,162	A80 vs. P40	2.1	58	2.62§	4,162 (100%)	328 (8%)	0 (0%)	911 (22%)	−0.65
A to Z	4,497	S40 then S80 vs. placebo then S20	2.0	60	2.09§	4,497 (100%)	479 (11%)	0 (0%)	1,100 (24%)	−0.30
TNT	10,001	A80 vs. A10	5.0	61	2.52	10,001 (100%)	1,537 (15%)	0 (0%)	1,902 (19%)	−0.62
IDEAL	8,888	A40-80 vs. S20-40	4.8	62	2.64§	8,888 (100%)	971 (11%)	0 (0%)	1,702 (19%)	−0.55
SEARCH	12,064	S80 vs. S20	7.0	64	2.50	12,064 (100%)	1,062 (9%)	0 (0%)	2,052 (17%)	−0.39
**Subtotal (5 trials)**	**39,612**	**-**	**5.1||**	**62||**	**2.53||**	**39,612 (100%)**	**4,377 (11%)**	**0 (0%)**	**7,667 (19%)**	**-0.51||**
**Total (27 trials)**	**174,149**	**-**	**4.9||**	**63||**	**-**	**92,280 (53%)**	**30,931 (18%)**	**70,025 (40%)**	**46,675 (27%)**	**-**

Trials are ordered by their date of publication. A = atorvastatin. F = fluvastatin. L = lovastatin. P = pravastatin. R = rosuvastatin. S = simvastatin. LDL-C = LDL cholesterol. CHD = coronary heart disease. 4D = Die Deutsche Diabetes Dialyse Studie. A to Z = Aggrastat to Zocor. AFCAPS/TexCAPS = Air Force/Texas Coronary Atherosclerosis Prevention Study. ALERT = Assessment of Lescol in Renal Transplantation. ALLHAT-LLT = Antihypertensive and Lipid-Lowering Treatment to Prevent Heart Attack Trial. ALLIANCE = Aggressive Lipid-Lowering Initiation Abates New Cardiac Events. ASCOT-LLA = Anglo-Scandinavian Cardiac Outcomes Trial–Lipid Lowering Arm. ASPEN = Atorvastatin Study for Prevention of Coronary Heart Disease Endpoints in Non-Insulin-Dependent Diabetes Mellitus. AURORA = A Study to Evaluate the Use of Rosuvastatin in Subjects on Regular Hemodialysis: An Assessment of Survival and Cardiovascular Events. CARDS = Collaborative Atorvastatin Diabetes Study. CARE = Cholesterol And Recurrent Events. GISSI-HF = Gruppo Italiano per lo Studio della Sopravvivenza nell'Insufficienza cardiac. GISSI–P = Gruppo Italiano per lo Studio della Sopravvivenza nell'Infarto Miocardico. HPS = Heart Protection Study. IDEAL = Incremental Decrease in End Points Through Aggressive Lipid Lowering Study Group. JUPITER = Justification for the Use of Statins in Prevention: an Intervention Trial Evaluating Rosuvastatin study group. LIPID = Long–term Intervention with Pravastatin in Ischaemic Disease. LIPS = Lescol Intervention Prevention Study. MEGA = Management of Elevated Cholesterol in the Primary Prevention Group of Adult Japanese Study Group. Post-CABG = Post-Coronary Artery Bypass Graft. PROSPER = PROspective Study of Pravastatin in the Elderly at Risk. PROVE-IT = Pravastatin or Atorvastatin Evaluation and Infection Therapy. SEARCH = Study of the Effectiveness of Additional Reductions in Cholesterol and Homocysteine. SSSS = Scandinavian Simvastatin Survival Study. TNT = Treating to New Targets. WOSCOPS = West of Scotland Coronary Prevention Study.

*Estimated with standard Kaplan-Meier methods, with patients censored at their date of death.

**History of MI or other symptomatic CHD.

† History of intracerebral bleed, transient ischaemic attack, ischaemic stroke, unknown stroke, peripheral artery disease or heart failure (if known).

†† Includes 382 randomised patients who were excluded from the trialists' primary publication.

|| Median follow–up, and mean age, baseline LDL-C and LDL-C difference at 1 year are weighted by the trial–specific variances of the observed logrank (o–e) statistic for major vascular events.

§ These three trials did not have active run–in periods; the values shown are the estimated on-treatment LDL cholesterol levels in the standard statin group.

### Cancers diagnosed after randomization

**Figure 1:**
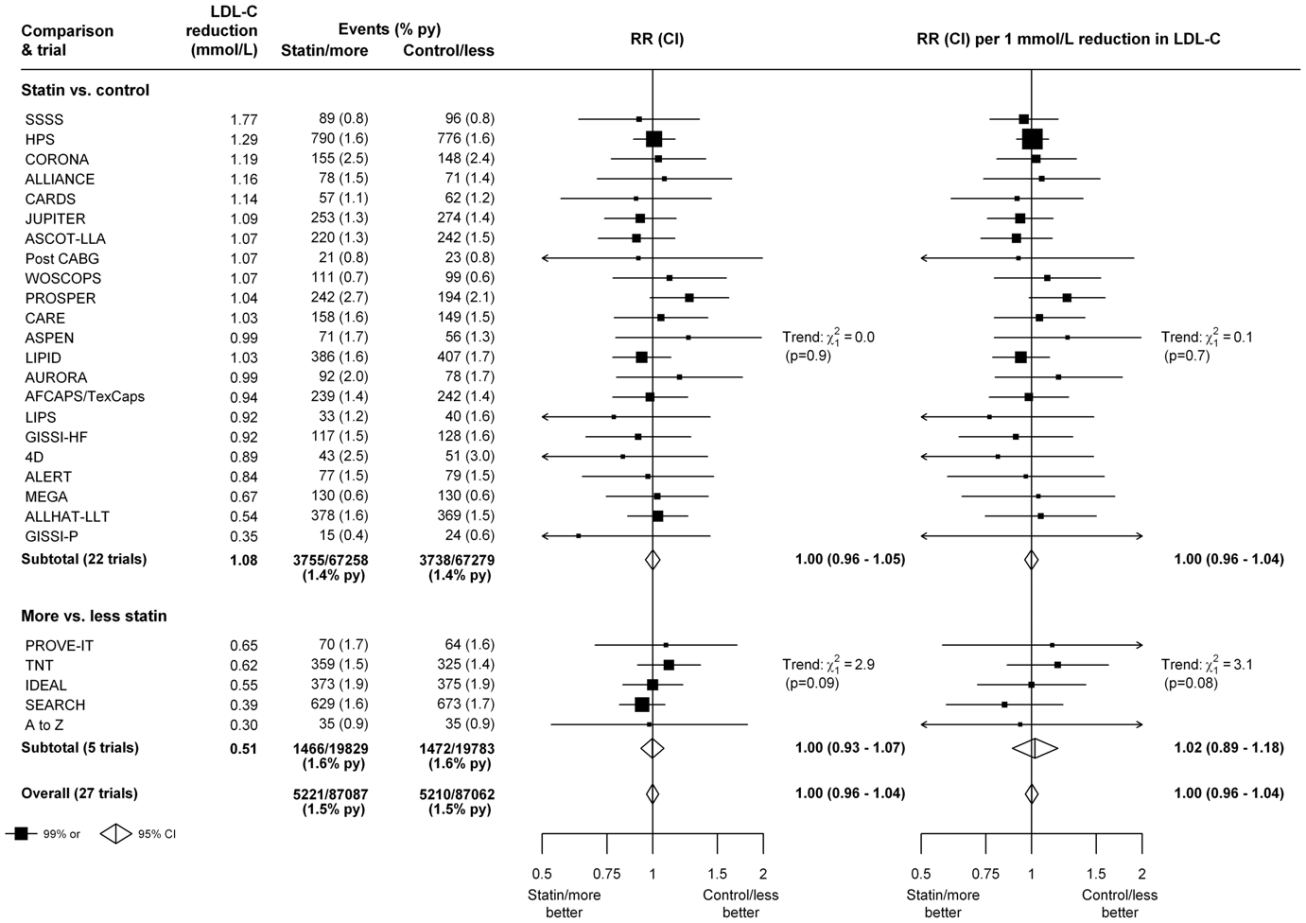
Effects of statin therapy on cancer incidence in each study. In the left panel, unweighted rate ratios (RRs) are plotted for each trial of the comparison of first event rates between randomly allocated treatment groups, along with their 99% confidence intervals (CIs). Trials are ordered according to the absolute reduction in LDL cholesterol at 1 year within each type of trial comparison (statin versus control and more versus less statin). In the right panel, rate ratios are weighted per 1 mmol/L LDL cholesterol difference at 1 year. Totals and subtotals, together with their 95% CIs, are indicated by open diamonds.

First cancers after randomization were recorded in the 22 trials of statin versus control among 3755 (1.4% per year [py]) of 67 258 participants allocated statin therapy versus 3738 (1.4% py) of 67 279 allocated control, corresponding to a rate ratio of 1.00 (95% CI 0.96–1.05), or an LDL-weighted rate ratio of 1.00 (95% CI 0.96–1.04) per 1 mmol/L LDL cholesterol reduction (Figure 1). In the 5 trials of more versus less intensive statin therapy, first cancers after randomization were recorded among 1466 (1.6% py) of 19 829 participants allocated more intensive versus 1472 (1.6% py) of 19 783 allocated less intensive therapy (Figure 1), corresponding to a rate ratio (RR) of 1.00 (95% CI 0.93–1.07), which was equivalent to an LDL-weighted rate ratio of 1.02 (95% CI 0.89–1.18) per 1 mmol/L LDL cholesterol reduction (Figure 1). Taking all 27 trials together, there was no evidence that lowering LDL cholesterol increased the overall incidence of cancer (RR 1.00, 95% CI 0.96–1.04).

**Figure 2:**
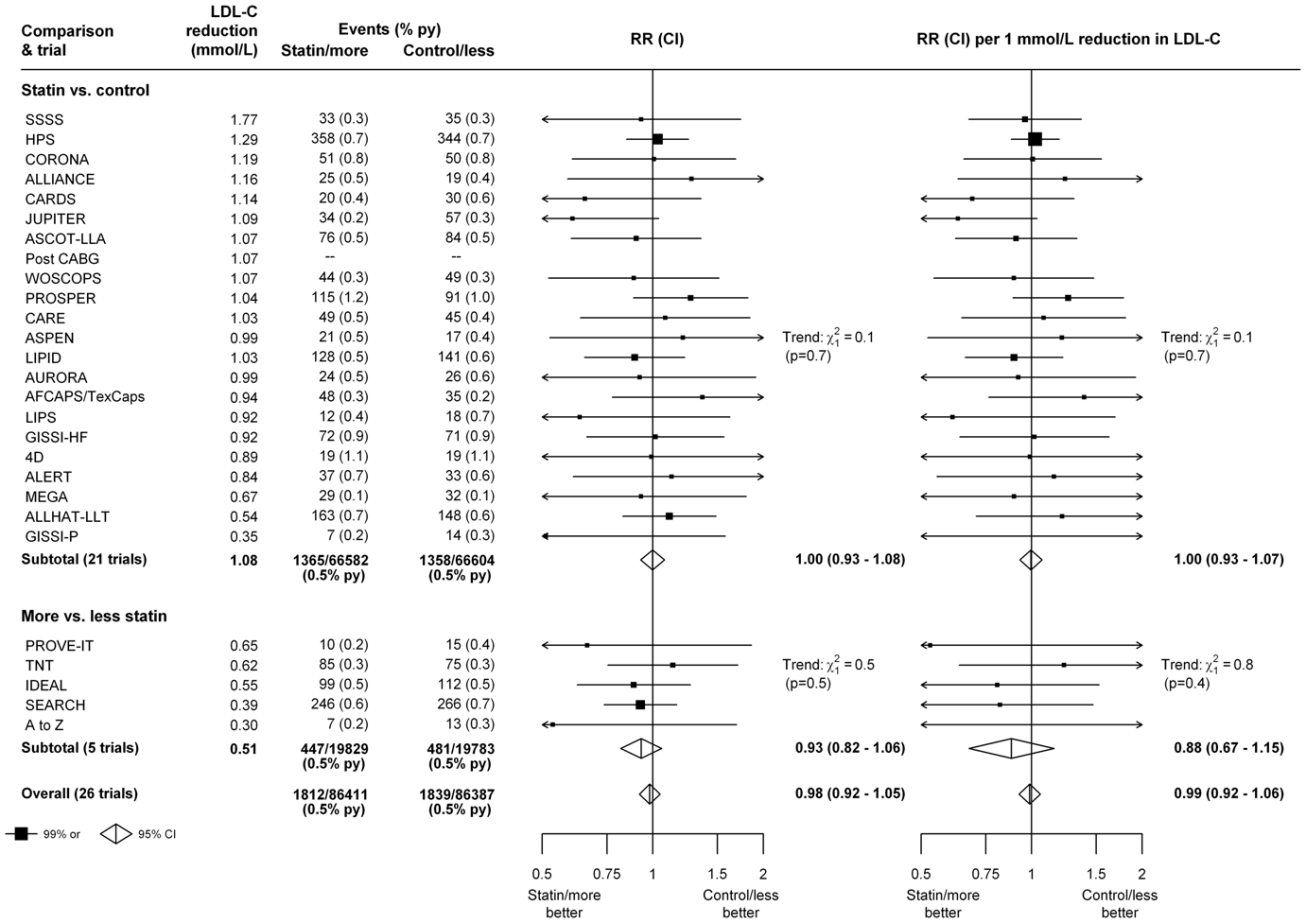
Effects of statin therapy on cancer mortality in each study. Symbols and conventions as in Figure 1. Deaths from cancers known to have been first diagnosed prior to randomization are excluded.

Likewise, there was no evidence of any excess in newly diagnosed cancers that resulted in death in either type of trial (Figure 2). Twenty one of the 22 trials of statin versus control provided information on cancer mortality. In these trials, 1365 patients allocated statin versus 1358 patients allocated control died from cancer (RR 1.00 [95% CI 0.93–1.08], or an LDL-weighted rate ratio of 1.00 [95% CI 0.93–1.07] per 1 mmol/L LDL cholesterol reduction), while in the 5 trials of more versus less intensive statin therapy, 447 patients allocated more intensive versus 481 patients allocated less intensive therapy died from cancer (RR 0.93 [95% CI 0.82–1.06], which was equivalent to an LDL-weighted rate ratio of 0.88 [95% CI 0.67–1.15] per 1 mmol/L LDL cholesterol reduction). Taking all trials together, there was no evidence that lowering LDL cholesterol increased cancer mortality (rate ratio 0.98 [95% CI 0.92–1.05]).

Since there was no significant heterogeneity among the results of the trials for either cancer incidence (Figure 1) or cancer mortality (Figure 2), and since the results weighted for LDL cholesterol differences between studies yielded virtually identical results to the unweighted analyses, subsequent analyses focus on the unweighted results seen in all 27 trials (separate analyses of the trials of statin versus control and the trials of more versus less statin can be found in the supporting information).

### Anatomical site of cancer

Although there was no evidence of an increase in the overall incidence of any cancer within the 27 trials of statin therapy, such an analysis would be insensitive to an increase in just one or a few types of cancer. There was, however, no evidence of an increased risk of cancer at any of 23 individual categories of sites, either in the 27 trials considered together (table 2), or separately in the 22 trials of statin versus control or the 5 trials of more versus less intensive statin therapy (Table S2). Similarly, there was no evidence of any increased risk of death from cancer at any individual site (table 2 and Table S2). (Note: The apparent reductions in liver cancer incidence [7 vs 18; nominal p = 0.05] and in deaths due to cancers from other known sites [5 vs 16; nominal p = 0.03] among the 5 trials of more versus less intensive statin therapy [Table S2] were not significant after adjustment for multiplicity.)

**Table 2:** Cancer incidence and cancer mortality in all 27 trials, by site.

	Cancer incidence			Cancer mortality*		
	Statin/More(n = 87087)	Control/Less(n = 87062)	p value	Statin/More(n = 87087)	Control/Less(n = 87062)	p value
Total follow-up (person years)	359581	358764		367936	367146	
**Site of Cancer**						
**Gastrointestinal**	**1214**	**1245**	**0.49**	**503**	**507**	**0.86**
Lip, mouth or pharynx	68	66	0.95	10	15	0.41
Oesophageal	81	83	0.92	45	55	0.36
Stomach	118	124	0.75	64	54	0.43
Large bowel or intestine	549	567	0.57	148	165	0.34
Liver	42	51	0.39	28	32	0.68
Gall bladder or bile-ducts	26	30	0.67	22	20	0.89
Pancreas	106	96	0.54	82	71	0.44
Other gastrointestinal	224	228	0.84	104	95	0.58
**Genitourinary**	**1644**	**1676**	**0.52**	**222**	**238**	**0.45**
Prostate	923	954	0.44	104	107	0.87
Penis/Scrotum	140	123	0.34	4	3	1.00
Uterus	57	60	0.86	7	8	0.99
Ovarian	35	36	1.00	14	16	0.86
Other genitourinary	17	14	0.73	5	2	0.45
Bladder	315	331	0.53	49	64	0.18
Kidney	157	158	0.97	39	38	1.00
**Respiratory**	**845**	**847**	**0.93**	**553**	**584**	**0.34**
Trachea/Lung	709	705	0.98	462	495	0.27
Other respiratory	136	142	0.74	91	89	0.95
**Female breast**	**273**	**244**	**0.22**	**24**	**17**	**0.35**
**Haematological**	**313**	**301**	**0.70**	**118**	**120**	**0.92**
**Melanoma**	**160**	**145**	**0.45**	**17**	**19**	**0.86**
**Other/unspecified**	**772**	**752**	**0.65**	**375**	**354**	**0.50**
Neurological	67	57	0.44	55	45	0.39
Other known site	219	199	0.34	94	81	0.36
Unspecified	486	496	0.74	226	228	0.91
**All cancer**	**5221**	**5210**	**0.96**	**1812**	**1839**	**0.57**

Excluding death from cancers known to have been first diagnosed prior to randomisation. ICD-9 cancer codes: Gastrointestinal (140–159); Lip, mouth or pharynx (140–149); Oesophageal (150); Stomach (151); Large bowel or intestine (152–154); Liver (155); Gall bladder or bile-ducts (156); Pancreas (157); Other gastrointestinal (158,159); Genitourinary (179–189); Prostate (185); Penis/Scrotum (187); Uterus (179,180,182); Ovarian (183); Other genitourinary (181,184,186); Bladder (188); Kidney (189); Respiratory (160–163,165); Trachea/Lung (162); Other respiratory (160,161,163,165); Female breast (174); Haematological (200–208); Melanoma (172); Other/unspecified ([Neurological (191,192); Other known site (164,170,171,175,176,190,193–195); Unspecified (196–199, 209)]); All cancer (140–209 excluding 173). If the ICD9 cause of death was 173 or 210–239 then both cancer incidence and cancer death was coded as unknown cancer. P-values are continuity corrected.

### Incidence of cancer over time

**Figure 3:**
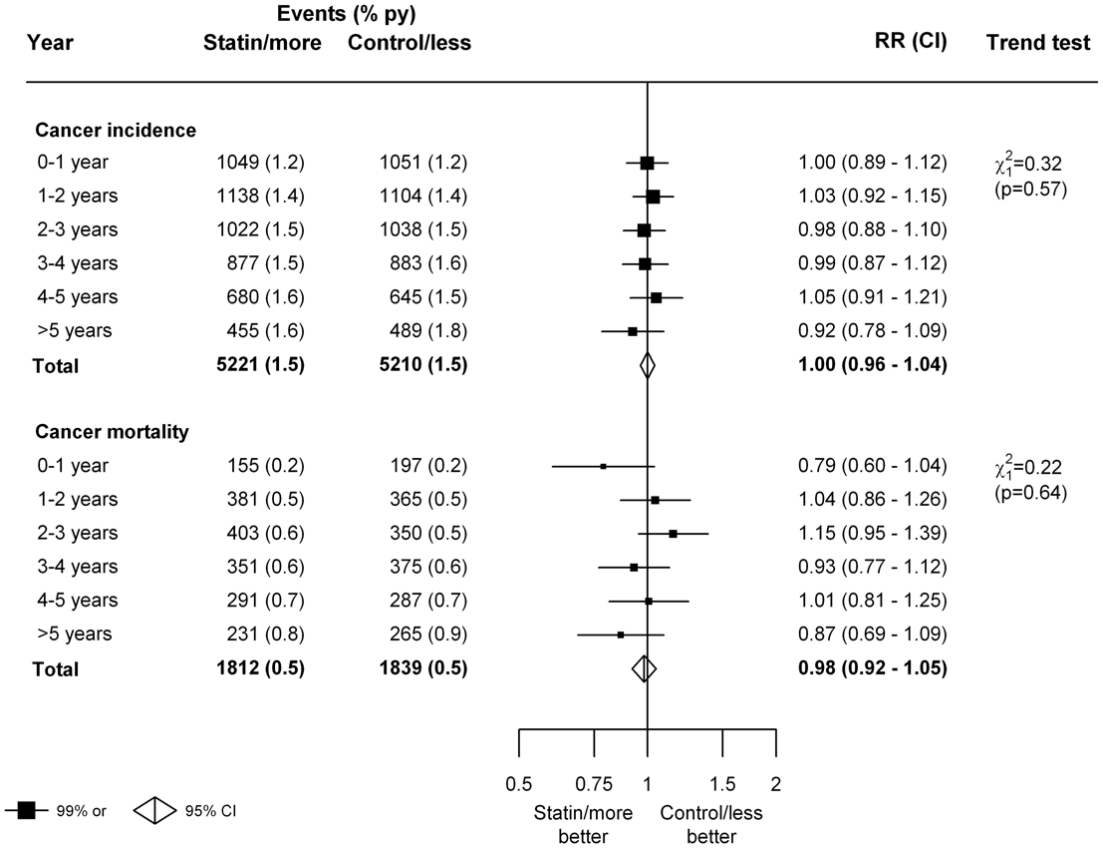
Effects of statin therapy on cancer incidence and mortality, by duration of treatment. Symbols and conventions as in Figure 1.

If lowering LDL cholesterol were a cause of cancer then it might be anticipated that the rate ratio for first cancers in each year of follow-up would tend to increase over time. There was, however, no evidence of a trend towards an increasing relative risk of a first cancer in all 27 trials (trend p = 0.57, Figure 3), or separately in the 22 trials of statin versus control or the 5 trials of more versus less statin (Figure S1). Similarly, there was no evidence of any such trends in analogous analyses of cancer mortality (trend p = 0.64 for all 27 trials: Figure 3 and Figure S2).

### LDL cholesterol before treatment

**Figure 4:**
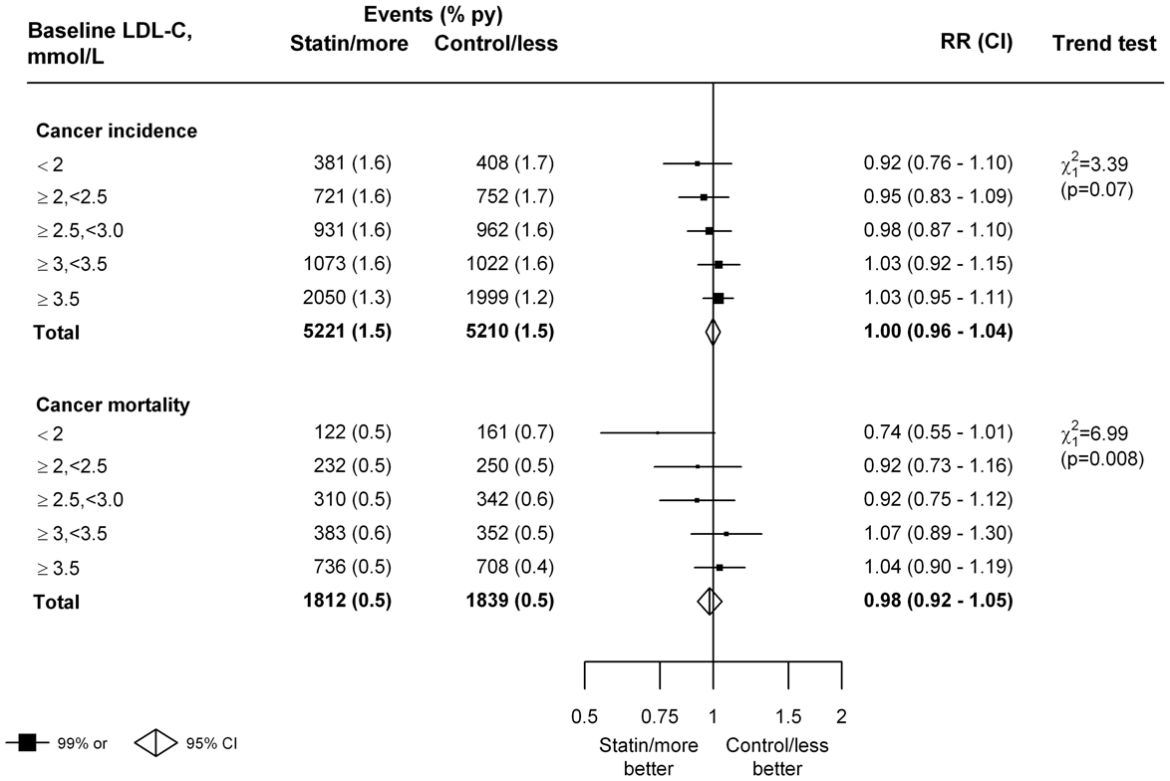
Effects of statin therapy on cancer incidence and mortality, by baseline LDL cholesterol. Symbols and conventions as in Figure 1. To convert from mmol/L to mg/dL divide by 0.02586.

If low LDL cholesterol concentration is a cause of cancer then one might expect to see a trend towards larger rate ratios among those with lower LDL cholesterol before treatment. However, if anything, there were fewer cancers among participants with lower baseline LDL cholesterol who were allocated statin or more intensive statin regimens (trend p = 0.07; Figure 4 and Figure S3). For instance, among individuals with baseline LDL cholesterol less than 2.00 mmol/L on the control or less intensive statin regimen, further LDL cholesterol reduction with a statin or more intensive statin regimen (from about 1.7 mmol/L to 1.3 mmol/L) was associated with a non-significant 8% reduction in cancer incidence (381 [1.6% py] versus 408 [1.7% py]; RR = 0.92, 99% CI 0.76–1.10) (Figure 4). In analyses of deaths due to cancer, a similar pattern was observed, with, if anything, smaller RRs observed among those with lower baseline LDL cholesterol levels (trend p = 0.008 for all 27 trials together; Figure 4 and Figure S4). These suggested trends were non-significant after adjustment for multiple testing however.

### Age, sex and other baseline characteristics

**Figure 5:**
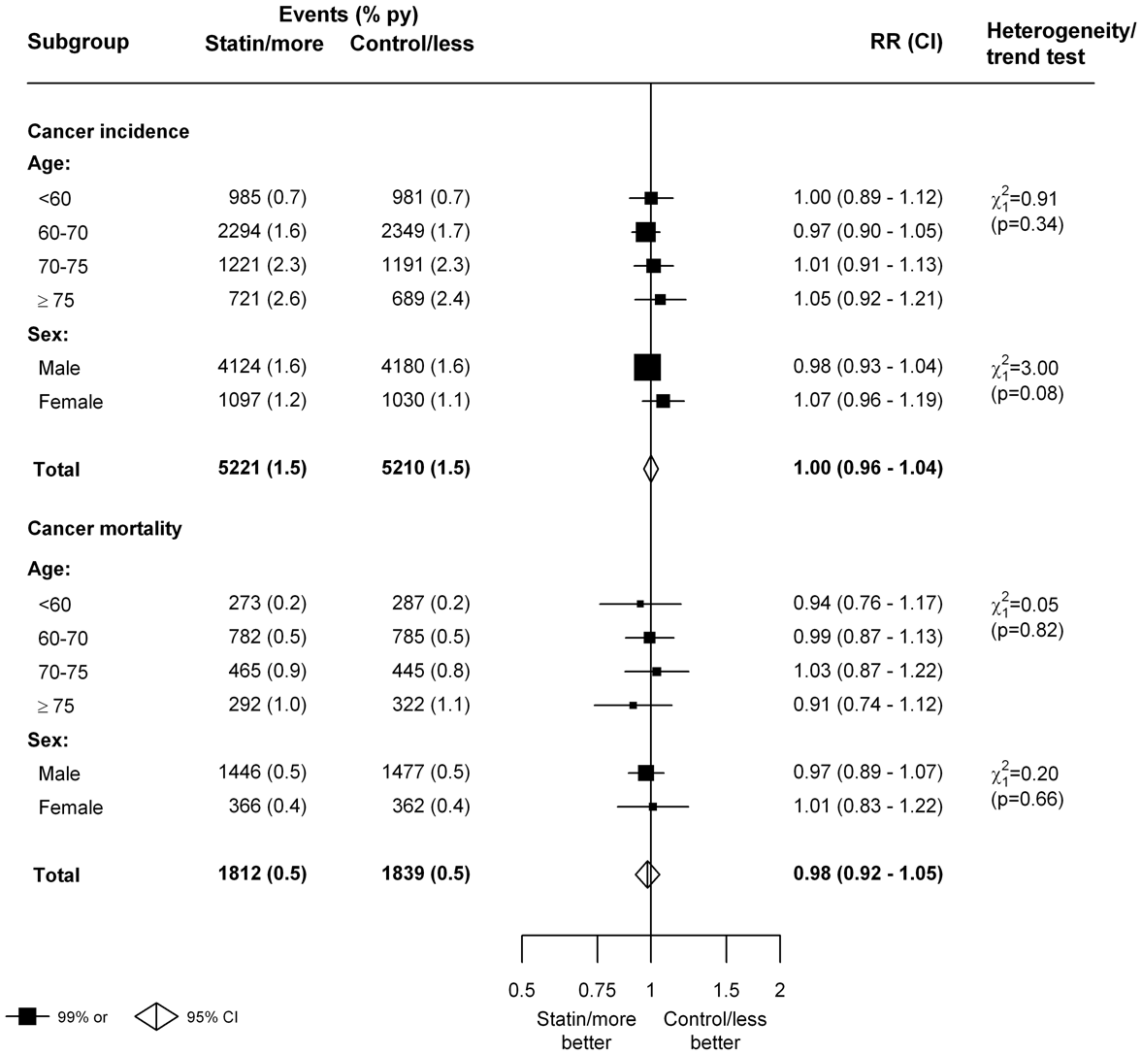
Effects of statin therapy on cancer incidence and mortality, by age and sex. Symbols and conventions as in Figure 1.

The PROSPER trial of pravastatin versus placebo conducted among people aged 70 or over had previously reported an excess risk of gastrointestinal cancer among statin allocated patients [10]. But, in the present meta-analysis, there was no evidence for an increased risk of any cancer among older people (Figure 5), even among those aged ≥75 at baseline (721 [2.6% py] statin/more statin versus 689 [2.4%] control/less statin; RR = 1.05, 99% CI 0.92–1.21) (Figure 5 and Figure S5). There was also no significant trend towards increasing rate ratios with older age (trend p = 0.34: Figure 5). Similarly, in analyses of cancer mortality, there was no evidence of any excess risk of death from cancer in older people and no evidence of an increasing trend in the rate ratio for cancer death with increasing age (Figure 5 and Figure S6). Rate ratios were also similar among men and women for both cancer incidence (heterogeneity p = 0.08; Figure 5) and for death from cancer (heterogeneity p = 0.66; Figure 5), and were also similar across a range of other baseline characteristics (Figures S7 and S8). (Note: The apparent trend towards a cancer excess among people with diabetes [heterogeneity p-value = 0.009: Figure S7] was not significant after adjustment for multiple testing.)

### Type of statin

In the 22 trials that compared statin therapy versus control, rate ratios for cancer incidence and death from cancer were similar irrespective of type of statin (Figures S9 and S10). In particular, there was no evidence that rate ratios differed between statins that are hydrophilic (pravastatin and rosuvastatin: cancer incidence RR 1.02 [95% CI 0.96 to 1.08]; cancer mortality RR 0.99 [95% CI 0.89 to 1.09]) and statins that are mostly lipophilic (atorvastatin, fluvastatin, lovastatin, simvastatin: cancer incidence RR 0.98 [95% CI 0.92 to 1.05]; cancer mortality RR 1.01 [95% CI 0.91 to 1.13]).

## Discussion

This meta-analysis of individual participant data from randomised trials provides reassuring evidence that reducing LDL cholesterol with statin therapy during a treatment period of about five years is not associated with an increased risk of developing a new cancer or of dying from cancer. In particular, it did not indicate any excess of particular types of cancer, or excesses of cancer with more prolonged or more intensive lowering of LDL cholesterol, even among older people. Nor was there any evidence that statin therapy reduces the risk of any particular type of cancer.

The findings of this meta-analysis are robust since they are based on over 10,000 cases of cancer and over 3500 deaths from cancer among 175,000 randomised patients. In addition, because they are derived from individual patient data, they provide a much more reliable test of the possible effects on cancer of lowering LDL cholesterol with a statin than has previously been possible from tabular meta-analyses. While individual patient data were not available from 2 eligible trials [19]; [20], their inclusion would have had no material effect on the findings: in one of those trials, 57 atorvastatin-allocated patients and 53 allocated placebo died from cancer (but the incidence of cancer was not reported) [19], while the other trial did not report the number of fatal or incident cancers but it included only 1600 patients [20].

Previously, it had been reported from observational studies in the general population [3] and from non-randomised analyses within statin trials [21]; [22], that lower levels of LDL cholesterol were associated with higher risks of cancer. The present meta-analysis of randomised evidence avoids the biases inherent in such non-randomised comparisons. Moreover, it involves large numbers of individuals in trials of more intensive statin therapy in whom LDL cholesterol was reduced to low levels. Consequently it is able to provide reliable evidence that there is no material cancer excess even when LDL cholesterol is reduced to about 1.3 mmol/L.

The present meta-analysis also provides reassurance that excesses in particular types of cancer observed in some of the individual trials were likely to have been due to the play of chance [23]. For example, the excess of breast cancer observed among women randomly allocated to pravastatin in the CARE trial (9 pravastatin versus 0 placebo; p = 0.004) [11], was not supported by the much larger number of female breast cancer cases in the other 26 trials (264 [1.1%] statin/more versus 244 [1.1%] control/less; p = 0.4). Similarly, the excess of gastrointestinal cancer originally reported in the PROSPER trial (65 pravastatin versus 45 placebo; p = 0.05) [10] was not supported by the results in the other trials shown here (1140 [1.4%] statin/more versus 1195 [1.4%] control/less; p = 0.2). It has also been suggested, based on observational and preclinical studies, that statins may prevent some types of cancer (such as prostate [24], oesophageal [25], colorectal [26]). But, again, this meta-analysis provides no evidence in support of such effects, at least within about five years of starting treatment.

An effect of lowering cholesterol on cancer risk might be missed if the latency period is substantially longer than the treatment period studied in these trials. There was, however, no suggestion in the meta-analysis of an increasing trend in the relative risk of cancer with increasing duration of treatment for up to about 6 years. Nor was there any suggestion in several of the individual trials that cancer risk increased during prolonged follow-up for up to a decade after the scheduled statin treatment period [27]-[30]. For example, in the WOSCOPS trial, no differences in cancer incidence were seen between the patients originally allocated pravastatin or placebo for 5 years during the subsequent 10 years [27] (reinforcing the results of 2 year post-trial follow-up in the LIPID trial [28]). Similarly, in the 4S trial, no significant differences in cancer incidence were seen between the patients allocated simvastatin or placebo for 5 years during the subsequent 5 years of follow-up [29]. More recently, 5-year post-trial follow-up of the 20,000 patients in the Heart Protection Study found no increased cancer risk associated with 5 years of prior treatment with simvastatin [30].

### Conclusion

It has been shown previously that reducing LDL cholesterol with a statin reduces the risk of major vascular events by about one-fifth for each 1 mmol/L reduction in LDL cholesterol, and that further reductions in LDL cholesterol with more intensive statin therapy produce further reductions in risk, even among patients who already have LDL cholesterol levels below 2 mmol/L [2]. The present report now demonstrates clearly that such reductions in LDL cholesterol do not increase the rate of cancer or cancer death, overall or at any particular site, during a treatment period of about 5 years (and more prolonged follow-up in some of the trials does not indicate any later excess) even among older individuals or those who have their cholesterol levels reduced to very low levels. These findings provide considerable reassurance about the safety of using more intensive statin regimens to lower LDL cholesterol levels substantially in patients who remain at high risk of major vascular events.

## Supporting Information

Figure S1 Effects of statin therapy on cancer incidence, by duration of treatment.
(PDF)

Figure S2 Effects of statin therapy on cancer mortality, by duration of treatment.
(PDF)

Figure S3 Effects of statin therapy on cancer incidence, by baseline LDL cholesterol.
(PDF)

Figure S4 Effects of statin therapy on cancer mortality, by baseline LDL cholesterol.
(PDF)

Figure S5 Effects of statin therapy on cancer incidence, by age and sex.
(PDF)

Figure S6 Effects of statin therapy on cancer mortality, by age and sex.
(PDF)

Figure S7 Effects of statin therapy on cancer incidence, by other baseline characteristics.
(PDF)

Figure S8 Effects of statin therapy on cancer mortality, by other baseline characteristics.
(PDF)

Figure S9 Effects of statin therapy on cancer incidence in 22 statin vs. control trials, by type of statin.
(PDF)

Figure S10 Effects of statin therapy on cancer mortality in 22 statin vs. control trials, by type of statin.
(PDF)

Table S1 Number of patients with a report of cancer (number of cancer deaths), by site and trial.
(PDF)

Table S2 Cancer incidence and cancer mortality by site, in 22 trials of statin vs. control and 5 trials of more vs. less statin.
(PDF)
