# Supplementary figures and images for: Lack of Effect of Lowering LDL Cholesterol on Cancer: Meta-Analysis of Individual Data from 175,000 People in 27 Randomised Trials of Statin Therapy
Source: PLoS One. 2012 Jan 19;7(1):e29849. doi: 10.1371/journal.pone.0029849 (PMC3261846; doi:10.1371/journal.pone.0029849)

**Figure S1: Effects of statin therapy on CANCER INCIDENCE, by duration of treatment**

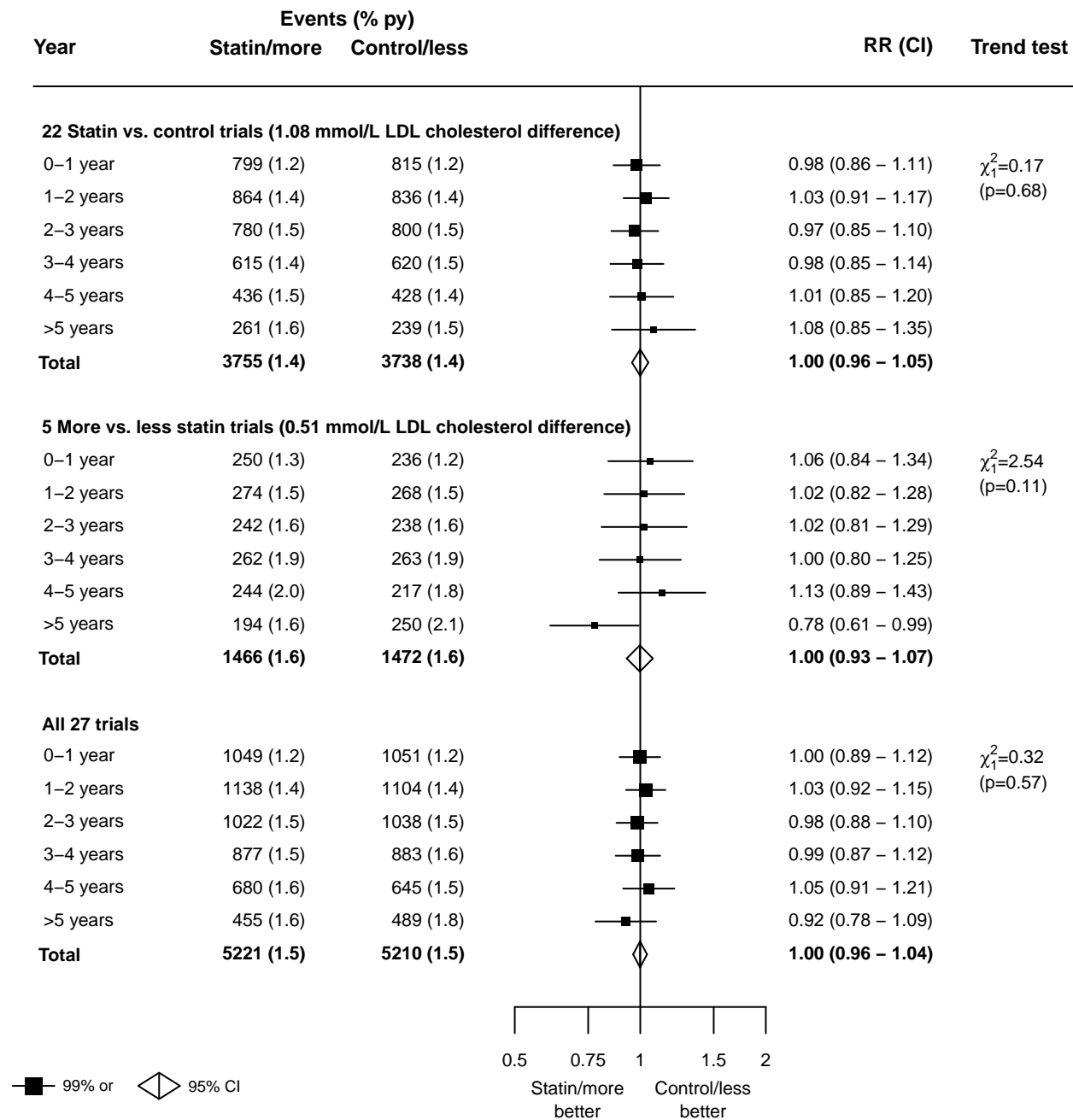

Supplement: Figure S1 — Effects of statin therapy on cancer incidence, by duration of treatment. (PDF) [file pone.0029849.s001.pdf]

**Figure S2: Effects of statin therapy on CANCER MORTALITY, by duration of treatment**

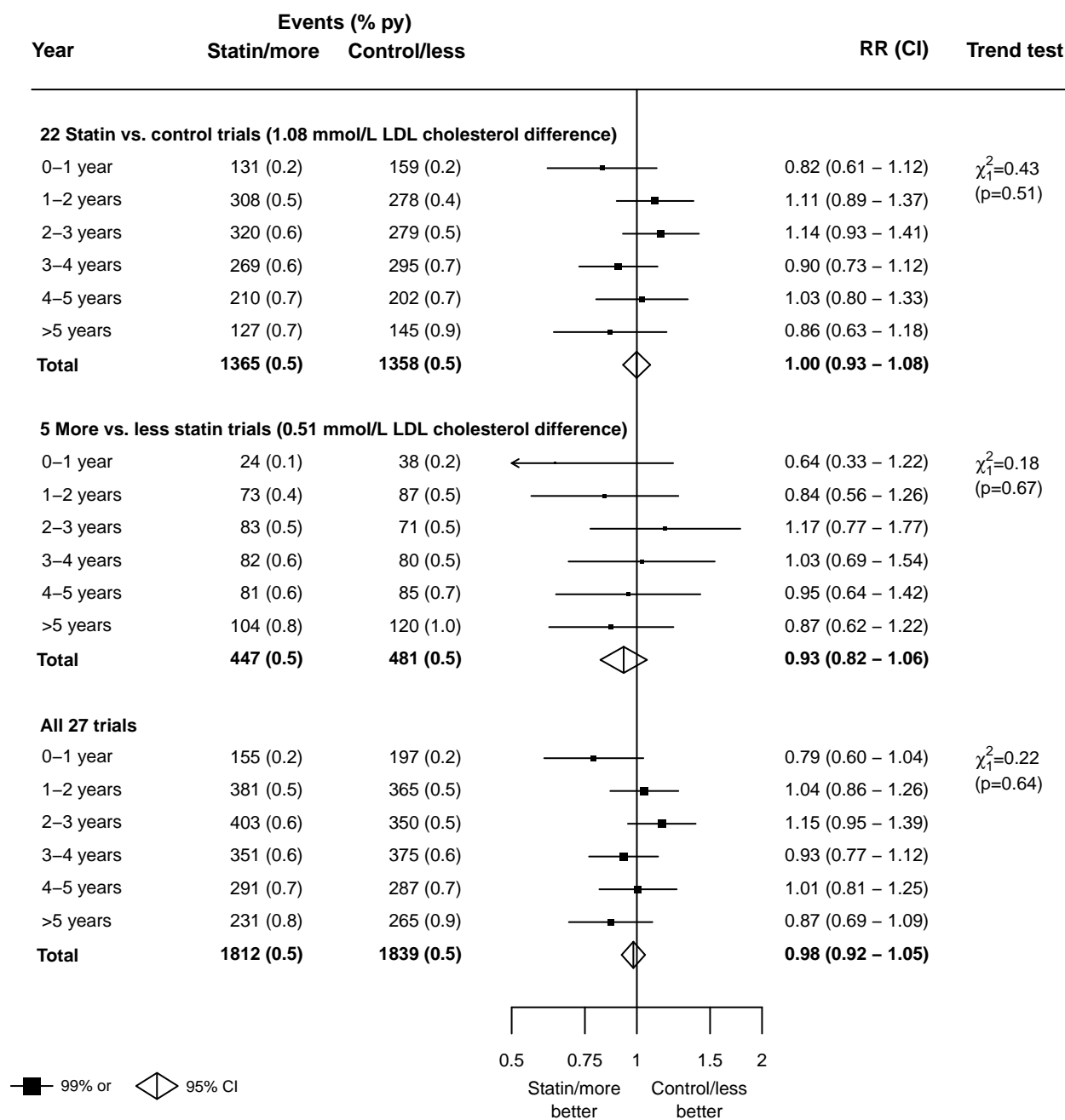

Supplement: Figure S2 — Effects of statin therapy on cancer mortality, by duration of treatment. (PDF) [file pone.0029849.s002.pdf]

**Figure S3: Effects of statin therapy on CANCER INCIDENCE, by baseline LDL cholesterol**

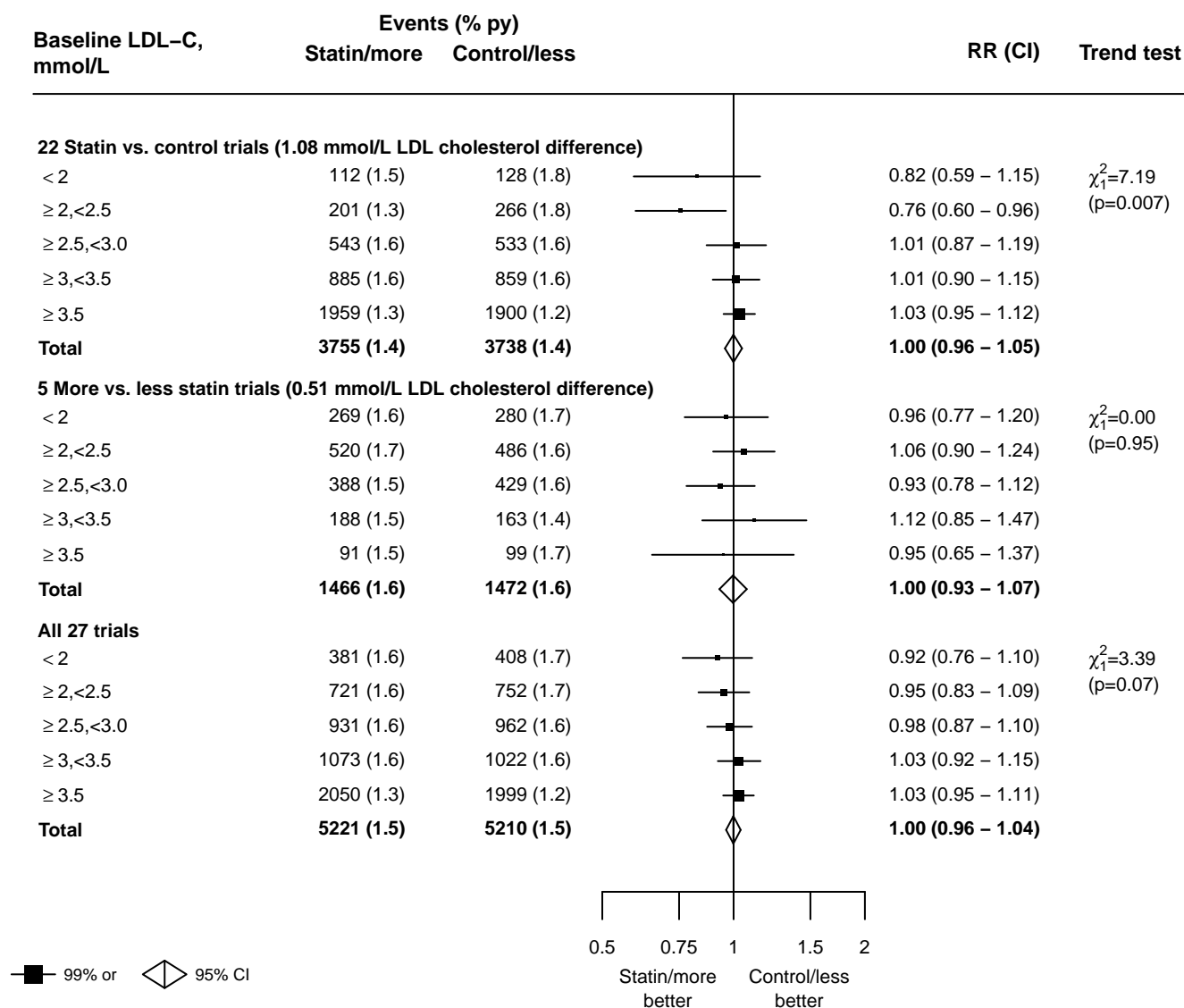

Supplement: Figure S3 — Effects of statin therapy on cancer incidence, by baseline LDL cholesterol. (PDF) [file pone.0029849.s003.pdf]

**Figure S4: Effects of statin therapy on CANCER MORTALITY, by baseline LDL cholesterol**

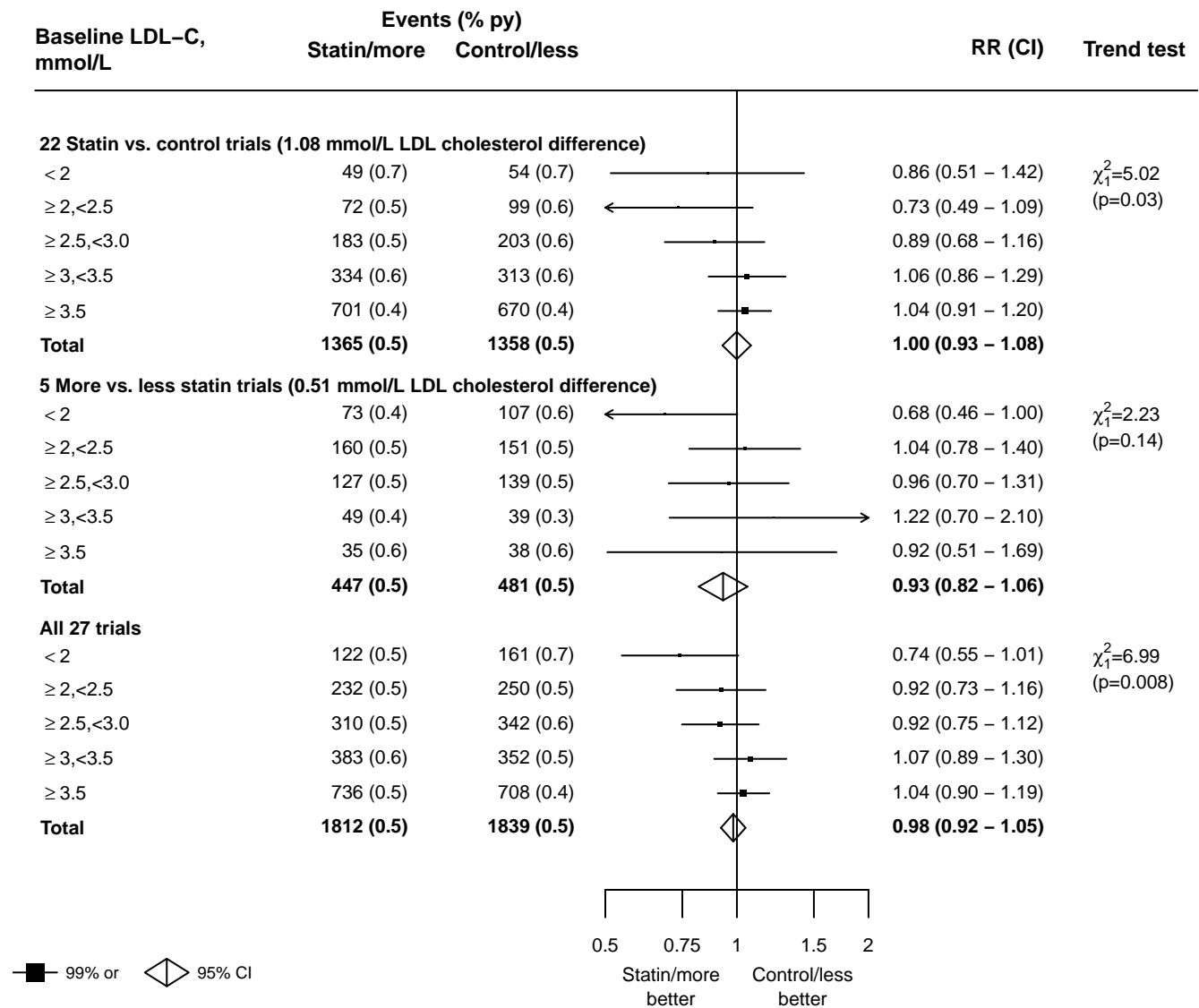

Supplement: Figure S4 — Effects of statin therapy on cancer mortality, by baseline LDL cholesterol. (PDF) [file pone.0029849.s004.pdf]

Figure S5: Effects of statin therapy on CANCER INCIDENCE, by age and sex

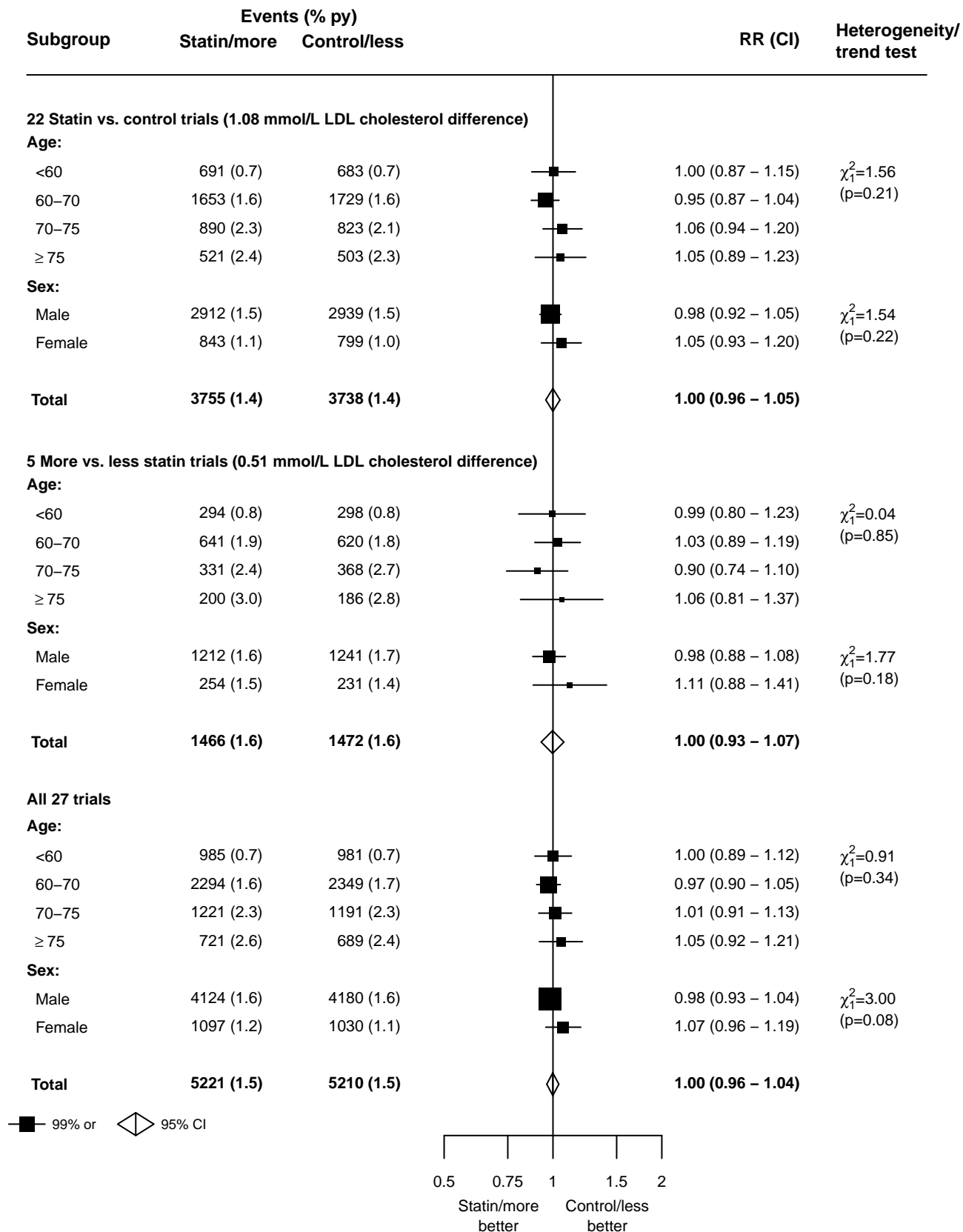

Supplement: Figure S5 — Effects of statin therapy on cancer incidence, by age and sex. (PDF) [file pone.0029849.s005.pdf]

**Figure S6: Effects of statin therapy on CANCER MORTALITY, by age and sex**

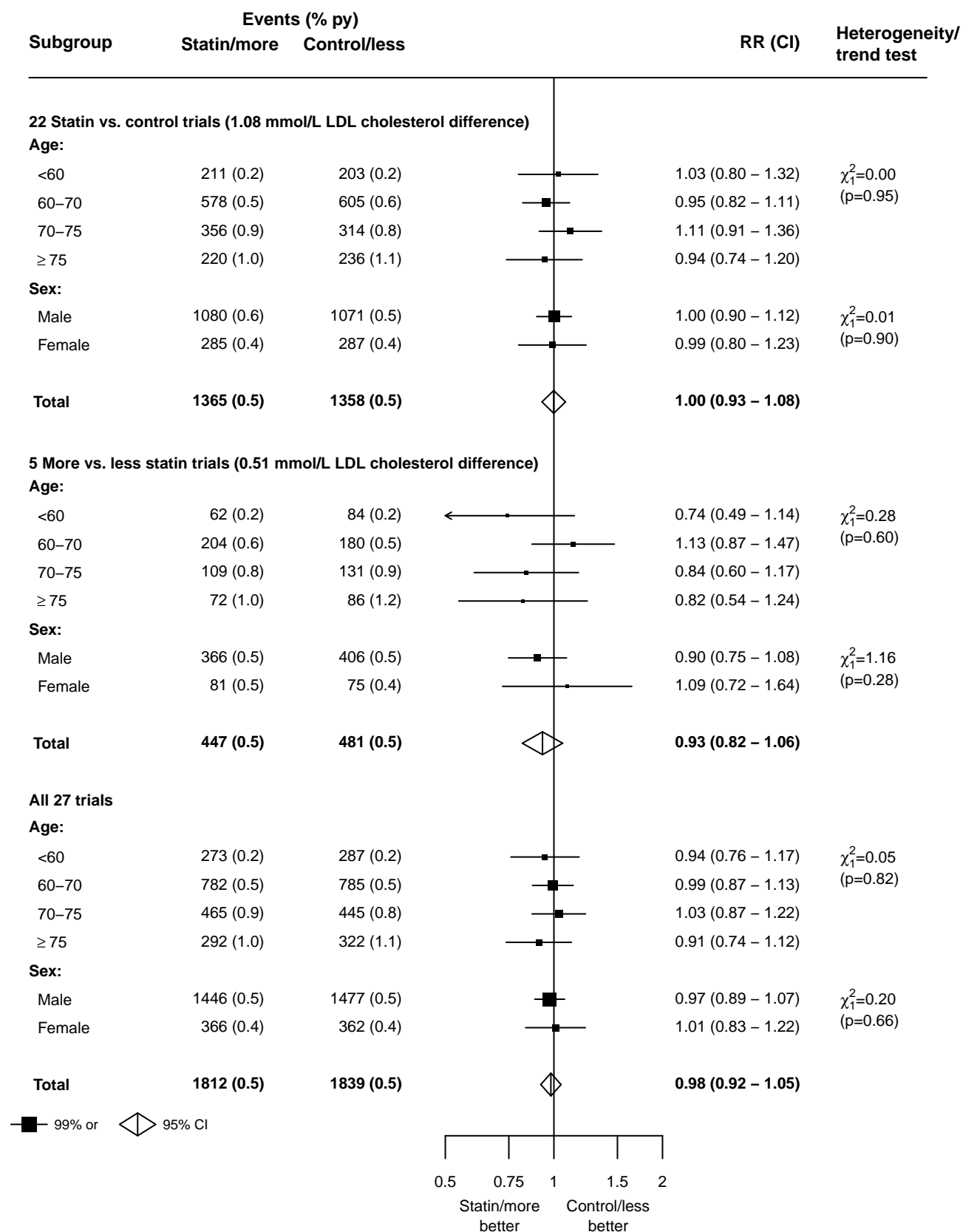

Supplement: Figure S6 — Effects of statin therapy on cancer mortality, by age and sex. (PDF) [file pone.0029849.s006.pdf]

**Figure S7: Effects of statin therapy on CANCER INCIDENCE, by other baseline characteristics**

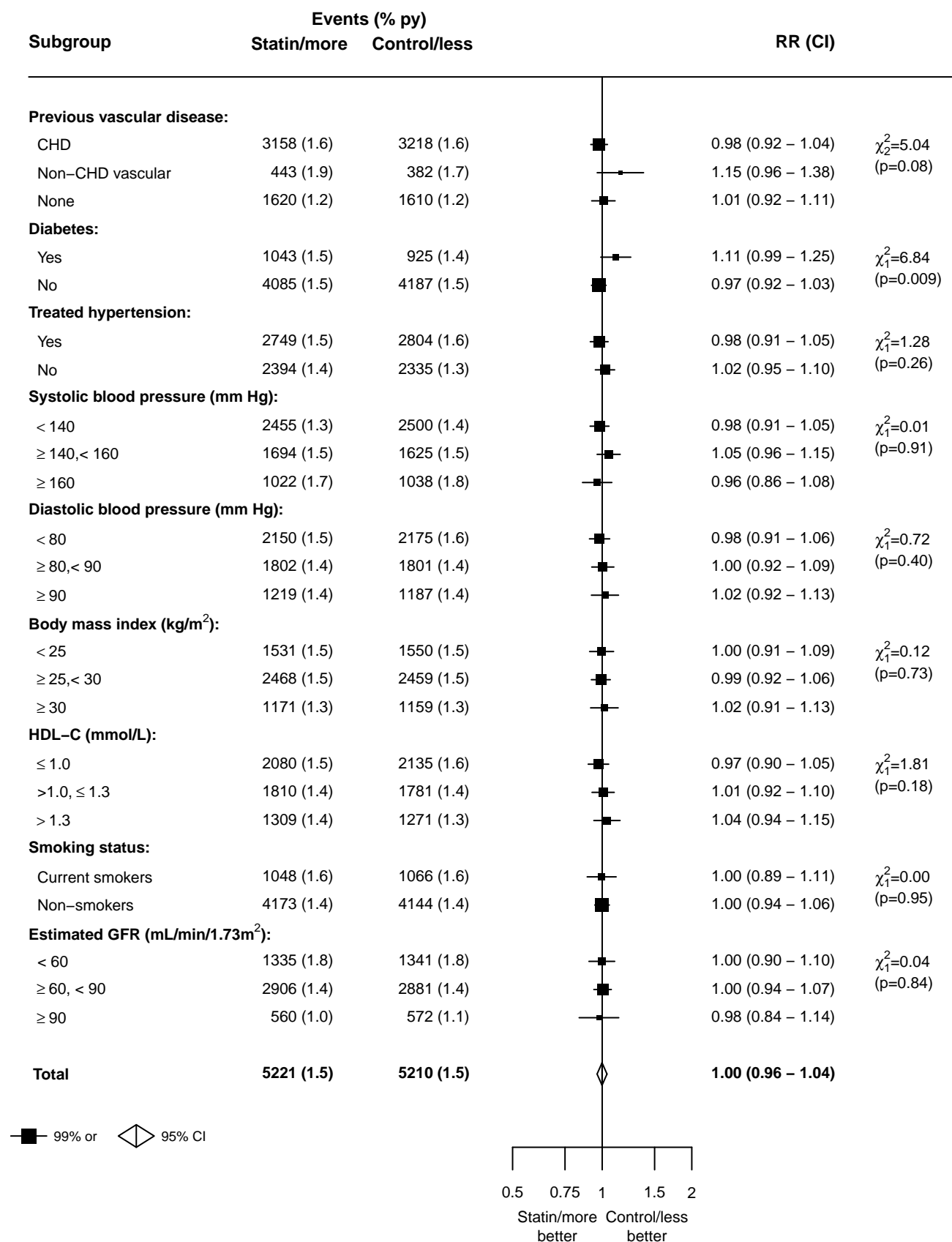

Supplement: Figure S7 — Effects of statin therapy on cancer incidence, by other baseline characteristics. (PDF) [file pone.0029849.s007.pdf]

**Figure S8: Effects of statin therapy on CANCER MORTALITY, by other baseline characteristics**

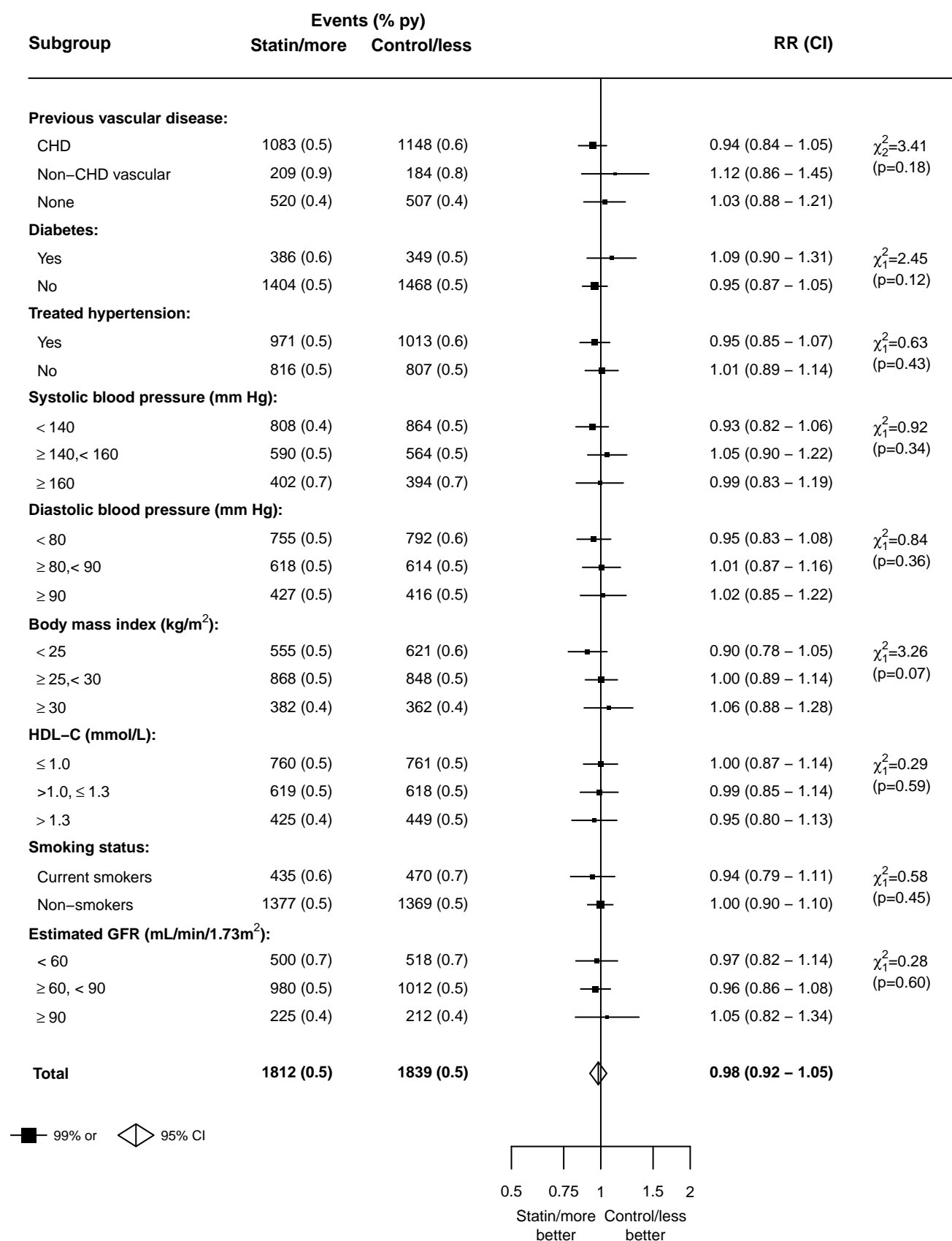

Supplement: Figure S8 — Effects of statin therapy on cancer mortality, by other baseline characteristics. (PDF) [file pone.0029849.s008.pdf]
