# Supplementary material for: Lack of Effect of Lowering LDL Cholesterol on Cancer: Meta-Analysis of Individual Data from 175,000 People in 27 Randomised Trials of Statin Therapy
Source: PLoS One. 2012 Jan 19;7(1):e29849. doi: 10.1371/journal.pone.0029849 (PMC3261846; doi:10.1371/journal.pone.0029849)
Supplement: Figure S9 — Effects of statin therapy on cancer incidence in 22 statin vs. control trials, by type of statin. (PDF) [file pone.0029849.s009.pdf]

Figure S9: Effects of statin therapy on CANCER INCIDENCE in 22 statin vs. control trials, by type of statin

Type of statin	Events (% py) Statin	Events (% py) Control	RR (CI)	Heterogeneity test
**Hydrophilic**				
Pravastatin	1420 (1.3)	1372 (1.3)	1.03 (0.94 − 1.14)	χ^2_1=0.62 (p=0.43)
Rosuvastatin	617 (1.6)	628 (1.6)	0.98 (0.85 − 1.13)	
**Subtotal:**	**2037 (1.4)**	**2000 (1.4)**	**1.02 (0.96 − 1.08)**	
**Lipophilic**				
Atorvastatin	469 (1.4)	482 (1.5)	0.96 (0.81 − 1.14)	χ^2_3=0.60 (p=0.90)
Fluvastatin	110 (1.4)	119 (1.5)	0.91 (0.64 − 1.27)	
Lovastatin	260 (1.3)	265 (1.4)	0.98 (0.78 − 1.22)	
Simvastatin	879 (1.4)	872 (1.4)	1.00 (0.88 − 1.13)	
**Subtotal:**	**1718 (1.4)**	**1738 (1.4)**	**0.98 (0.92 − 1.05)**	
**Total**	**3755 (1.4)**	**3738 (1.4)**	**1.00 (0.96 − 1.05)**	

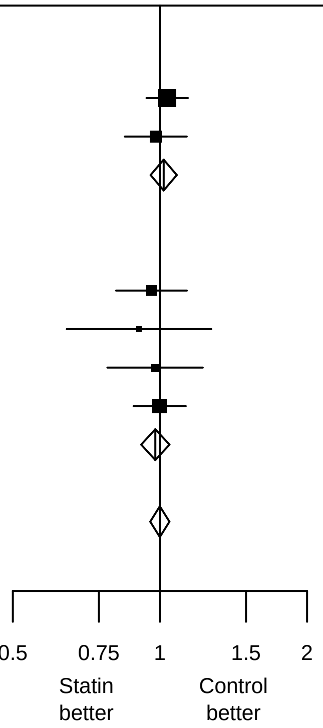

99% or   95% CI

Heterogeneity between hydrophilic and lipophilic statins: χ^2_1 = 0.72 (p=0.40)
