# Supplementary material for: Lack of Effect of Lowering LDL Cholesterol on Cancer: Meta-Analysis of Individual Data from 175,000 People in 27 Randomised Trials of Statin Therapy
Source: PLoS One. 2012 Jan 19;7(1):e29849. doi: 10.1371/journal.pone.0029849 (PMC3261846; doi:10.1371/journal.pone.0029849)
Supplement: Figure S10 — Effects of statin therapy on cancer mortality in 22 statin vs. control trials, by type of statin. (PDF) [file pone.0029849.s010.pdf]

Figure S10: Effects of statin therapy on CANCER MORTALITY in 22 statin vs. control trials, by type of statin

Type of statin	Events (% py) Statin	Events (% py) Control	RR (CI)	Heterogeneity test
**Hydrophilic**				
Pravastatin	535 (0.5)	520 (0.5)	1.03 (0.88 − 1.20)	χ^2_1=1.56 (p=0.21)
Rosuvastatin	181 (0.5)	204 (0.5)	0.89 (0.68 − 1.15)	
**Subtotal:**	**716 (0.5)**	**724 (0.5)**	**0.99 (0.89 − 1.09)**	
**Lipophilic**				
Atorvastatin	161 (0.5)	169 (0.5)	0.94 (0.71 − 1.25)	χ^2_3=2.43 (p=0.49)
Fluvastatin	49 (0.6)	51 (0.6)	0.95 (0.57 − 1.58)	
Lovastatin	48 (0.2)	35 (0.2)	1.36 (0.78 − 2.40)	
Simvastatin	391 (0.6)	379 (0.6)	1.02 (0.85 − 1.23)	
**Subtotal:**	**649 (0.5)**	**634 (0.5)**	**1.01 (0.91 − 1.13)**	
**Total**	**1365 (0.5)**	**1358 (0.5)**	**1.00 (0.93 − 1.08)**	

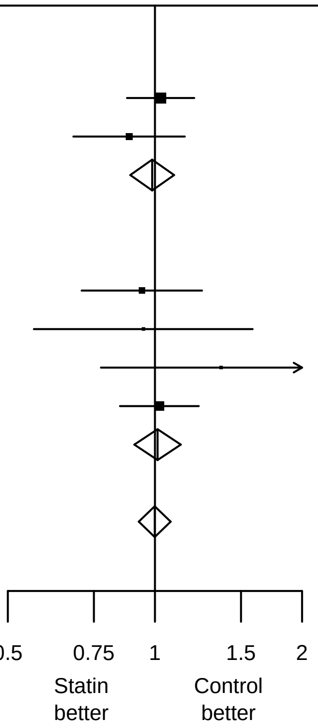

99% or 95% CI

Heterogeneity between hydrophilic and lipophilic statins: χ^2_1 = 0.11 (p=0.74)
