# Supplementary material for: Lack of Effect of Lowering LDL Cholesterol on Cancer: Meta-Analysis of Individual Data from 175,000 People in 27 Randomised Trials of Statin Therapy
Source: PLoS One. 2012 Jan 19;7(1):e29849. doi: 10.1371/journal.pone.0029849 (PMC3261846; doi:10.1371/journal.pone.0029849)
Supplement: Table S1 — Number of patients with a report of cancer (number of cancer deaths), by site and trial. (PDF) [file pone.0029849.s011.pdf]

Table S1: Number of patients with a report of cancer (number of cancer deaths), by site and trial

Study	Any cancer	Gastrointestinal cancer	Genitourinary cancer	Respiratory cancer	Female Breast cancer	Haematological cancer	Melanoma	Neurological cancer	Other known site	Unspecified
**Statin vs. control**										
SSSS	185 (68)	52 (22)	61 (10)	31 (22)	8 (1)	16 (8)	11 (2)	1 (1)	3 (1)	2 (1)
WOSCOPS	210 (93)	76 (32)	58 (9)	45 (37)	0 (0)	9 (3)	0 (0)	7 (5)	6 (0)	9 (7)
CARE	307 (94)	64 (25)	132 (6)	60 (44)	9 (0)	22 (8)	7 (0)	6 (6)	4 (3)	3 (2)
Post CABG	44 (0)	2 (0)	30 (0)	3 (0)	1 (0)	0 (0)	0 (0)	0 (0)	0 (0)	8 (0)
AFCAPS/TexCaps	481 (83)	61 (21)	243 (8)	47 (26)	21 (1)	46 (13)	42 (3)	6 (6)	9 (1)	6 (4)
LIPID	793 (269)	172 (68)	305 (33)	115 (78)	17 (0)	56 (22)	52 (8)	16 (14)	11 (3)	49 (43)
GISSI-P	39 (21)	9 (5)	8 (2)	13 (10)	1 (0)	5 (3)	1 (0)	1 (0)	1 (1)	0 (0)
LIPS	73 (30)	22 (10)	18 (4)	16 (7)	3 (0)	5 (0)	0 (0)	3 (3)	4 (4)	2 (2)
HPS	1566 (702)	441 (217)	506 (100)	328 (257)	83 (10)	91 (40)	26 (4)	15 (12)	12 (2)	64 (60)
PROSPER	436 (206)	124 (65)	115 (23)	88 (65)	25 (0)	19 (8)	11 (1)	4 (4)	7 (7)	43 (33)
ALLHAT-LLT	747 (311)	83 (30)	197 (27)	135 (92)	70 (11)	0 (0)	0 (0)	0 (0)	228 (122)	34 (29)
ASCOT-LLA	462 (160)	119 (57)	158 (25)	57 (31)	12 (1)	36 (17)	5 (2)	8 (5)	10 (0)	57 (22)
ALERT	156 (70)	27 (15)	40 (6)	16 (11)	15 (2)	22 (11)	7 (2)	2 (2)	8 (4)	19 (17)
CARDS	119 (50)	30 (10)	23 (4)	12 (5)	14 (0)	4 (1)	2 (0)	1 (1)	2 (1)	31 (28)
ALLIANCE	149 (44)	9 (3)	13 (1)	10 (10)	3 (0)	2 (1)	0 (0)	1 (0)	0 (0)	111 (29)
4D	94 (38)	32 (20)	13 (2)	7 (4)	8 (1)	6 (2)	3 (1)	0 (0)	4 (0)	21 (8)
ASPEN	127 (38)	30 (11)	48 (4)	17 (12)	7 (0)	3 (1)	6 (1)	0 (0)	3 (0)	13 (9)
MEGA	260 (61)	128 (33)	55 (9)	23 (9)	24 (0)	11 (3)	0 (0)	3 (1)	10 (0)	6 (6)
GISSI-HF	245 (143)	91 (50)	40 (12)	58 (48)	7 (0)	0 (0)	1 (0)	10 (9)	0 (0)	38 (24)
JUPITER	527 (91)	135 (31)	161 (8)	72 (27)	44 (1)	50 (8)	25 (2)	6 (3)	18 (4)	16 (7)
AURORA	170 (50)	46 (16)	46 (8)	28 (12)	9 (2)	14 (3)	5 (0)	2 (2)	7 (0)	13 (7)
CORONA	303 (101)	96 (34)	74 (12)	61 (27)	9 (0)	20 (10)	3 (0)	2 (2)	7 (1)	31 (15)
**Subtotal: All 22 statin vs. control trials**	**7493 (2723)**	**1849 (775)**	**2344 (313)**	**1242 (834)**	**390 (30)**	**437 (162)**	**207 (26)**	**94 (76)**	**354 (154)**	**576 (353)**
**More vs. less statin**										
PROVE-IT	134 (25)	39 (5)	32 (1)	18 (11)	9 (0)	7 (0)	0 (0)	1 (1)	15 (2)	13 (5)
A to Z	70 (20)	20 (7)	21 (4)	16 (4)	3 (0)	0 (0)	0 (0)	1 (1)	2 (1)	7 (3)
TNT	684 (160)	115 (33)	280 (18)	82 (52)	30 (1)	39 (21)	29 (0)	2 (0)	11 (7)	96 (28)
IDEAL	748 (211)	125 (57)	190 (38)	101 (72)	37 (2)	25 (14)	14 (3)	9 (8)	13 (1)	234 (16)
SEARCH	1302 (512)	311 (133)	453 (86)	233 (164)	48 (8)	106 (41)	55 (7)	17 (14)	23 (10)	56 (49)
**Subtotal: All 5 more vs. less trials**	**2938 (928)**	**610 (235)**	**976 (147)**	**450 (303)**	**127 (11)**	**177 (76)**	**98 (10)**	**30 (24)**	**64 (21)**	**406 (101)**
**Total**	**10431 (3651)**	**2459 (1010)**	**3320 (460)**	**1692 (1137)**	**517 (41)**	**614 (238)**	**305 (36)**	**124 (100)**	**418 (175)**	**982 (454)**

ICD-9 cancer codes: All cancer (140-209 excluding 173); Gastrointestinal (140-159); Genitourinary (179-189); Respiratory (160-163,165); Female breast (174); Haematological (200-208); Melanoma (172); Neurological (191,192); Other known site (164,170,171,175,176,190,193-195); Unspecified (196-199, 209). If the ICD9 cause of death was 173 or 210-239 then both cancer incidence and cancer death was coded as unknown cancer.
