# Supplementary material for: Lack of Effect of Lowering LDL Cholesterol on Cancer: Meta-Analysis of Individual Data from 175,000 People in 27 Randomised Trials of Statin Therapy
Source: PLoS One. 2012 Jan 19;7(1):e29849. doi: 10.1371/journal.pone.0029849 (PMC3261846; doi:10.1371/journal.pone.0029849)
Supplement: Table S2 — Cancer incidence and cancer mortality by site, in 22 trials of statin vs. control and 5 trials of more vs. less statin. (PDF) [file pone.0029849.s012.pdf]

Table S2: Cancer incidence and cancer mortality by site, in 22 trials of statin vs. control and 5 trials of more vs. less statin

	Statin vs. control (22 trials)						More vs. Less statin (5 trials)					
	Cancer incidence			Cancer mortality			Cancer incidence			Cancer mortality		
	Statin (n=67258)	Control (n=67279)	Nominal p value	Statin (n=67258)	Control (n=67279)	Nominal p value	More (n=19829)	Less (n=19783)	Nominal p value	More (n=19829)	Less (n=19783)	Nominal p value
Total follow-up (person years)	268847	267933		274479	273707		90734	90830		93456	93439	
**Site of Cancer**												
**Gastrointestinal**	**926**	**923**	**0.99**	**395**	**380**	**0.68**	**288**	**322**	**0.18**	**108**	**127**	**0.24**
Lip, mouth or pharynx	49	52	0.82	8	13	0.38	19	14	0.49	2	2	1.00
Oesophageal	61	62	0.97	33	39	0.54	20	21	1.00	12	16	0.57
Stomach	92	100	0.61	50	45	0.71	26	24	0.89	14	9	0.40
Large bowel or intestine	445	448	0.89	124	134	0.54	104	119	0.35	24	31	0.41
Liver	35	33	0.93	24	24	1.00	7	18	0.05	4	8	0.39
Gall bladder or bile-ducts	19	21	0.86	17	14	0.74	7	9	0.80	5	6	1.00
Pancreas	84	74	0.49	66	52	0.25	22	22	1.00	16	19	0.74
Other gastrointestinal	141	133	0.72	73	59	0.27	83	95	0.41	31	36	0.63
**Genitourinary**	**1164**	**1180**	**0.66**	**145**	**168**	**0.19**	**480**	**496**	**0.65**	**77**	**70**	**0.62**
Prostate	671	685	0.64	65	76	0.38	252	269	0.49	39	31	0.40
Penis/Scrotum	72	58	0.28	4	3	1.00	68	65	0.85	0	0	n/a
Uterus	51	46	0.68	6	8	0.78	6	14	0.12	1	0	1.00
Ovarian	29	30	1.00	10	12	0.83	6	6	1.00	4	4	1.00
Other genitourinary	12	13	0.99	2	1	0.99	5	1	0.22	3	1	0.62
Bladder	211	228	0.41	34	41	0.46	104	103	0.99	15	23	0.25
Kidney	118	120	0.91	24	27	0.75	39	38	1.00	15	11	0.55
**Respiratory**	**614**	**628**	**0.65**	**404**	**430**	**0.34**	**231**	**219**	**0.60**	**149**	**154**	**0.82**
Trachea/Lung	562	559	1.00	384	405	0.43	147	146	1.00	78	90	0.40
Other respiratory	52	69	0.13	20	25	0.52	84	73	0.42	71	64	0.60
**Female breast**	**200**	**190**	**0.67**	**18**	**12**	**0.36**	**73**	**54**	**0.11**	**6**	**5**	**0.99**
**Haematological**	**218**	**219**	**0.94**	**78**	**84**	**0.66**	**95**	**82**	**0.36**	**40**	**36**	**0.72**
**Melanoma**	**104**	**103**	**1.00**	**13**	**13**	**1.00**	**56**	**42**	**0.19**	**4**	**6**	**0.75**
**Other/unspecified**	**529**	**495**	**0.34**	**312**	**271**	**0.11**	**243**	**257**	**0.58**	**63**	**83**	**0.11**
Neurological	53	41	0.27	43	33	0.32	14	16	0.86	12	12	1.00
Other known site	190	164	0.18	89	65	0.06	29	35	0.53	5	16	0.03
Unspecified	286	290	0.83	180	173	0.81	200	206	0.83	46	55	0.42
**All cancer**	**3755**	**3738**	**0.99**	**1365**	**1358**	**0.99**	**1466**	**1472**	**0.96**	**447**	**481**	**0.28**

ICD-9 cancer codes: Gastrointestinal (140-159); Lip, mouth or pharynx (140-149); Oesophageal (150); Stomach (151); Large bowel or intestine (152-154); Liver (155); Gall bladder or bile-ducts (156); Pancreas (157); Other gastrointestinal (158,159); Genitourinary (179-189); Prostate (185); Penis/Scrotum (187); Uterus (179,180,182); Ovarian (183); Other genitourinary (181,184,186); Bladder (188); Kidney (189); Respiratory (160-163,165); Trachea/Lung (162); Other respiratory (160,161,163,165); Female breast (174); Haematological (200-208); Melanoma (172); Other/unspecified ([Neurological (191,192); Other known site (164,170,171,175,176,190,193-195); Unspecified (196-199, 209)]); All cancer (140-209 excluding 173). If the ICD9 cause of death was 173 or 210-239 then both cancer incidence and cancer death was coded as unknown cancer.
